# Anti-Cancer Treatment Strategies in the Older Population: Time to Test More?

**DOI:** 10.3390/geriatrics6020042

**Published:** 2021-04-15

**Authors:** Antonino C. Tralongo, Roberto S. Fratamico, Chiara Russo, Andrea Sbrana, Andrea Antonuzzo, Marco Danova

**Affiliations:** 1 Medical Oncology Unit, Ospedale di Circolo e Fondazione Macchi, ASST dei Sette Laghi, Università di Pavia, 21100 Varese, Italy; 2 John and Dorothy Morgan Cancer Center, Lehigh Valley Cancer Institute, Allentown, PA 18103, USA; roberto.fratamico@lvhn.org; 3 Department of Medical Oncology, Centre Léon Bérard, Regional Comprehensive Cancer Centre, 69008 Lyon, France; Chiara.RUSSO@lyon.unicancer.fr; 4 Pulmonary Oncology Unit, AOU Pisana, 56126 Pisa, Italy; andreasbrana89@gmail.com; 5 Medical Oncology 2, AOU Pisana, 56126 Pisa, Italy; automezzo69@gmail.com; 6 Department of Internal Medicine and Medical Oncology, ASST di Pavia, 27029 Pavia, Italy; marco_danova@asst-pavia.it

**Keywords:** older patients, cancer, immunosenesce, metronomic chemotherapy, immunotherapy

## Abstract

Aging is a well-recognized risk factor for the development of cancer. The incidence of new cancer diagnoses has increased globally given the rising senior population. Many hypotheses for this increased risk have been postulated over decades, including increased genetic and epigenetic mutations and the concept of immunosenescence. The optimal treatment strategies for this population with cancer are unclear. Older cancer patients are traditionally under-represented in clinical trials developed to set the standard of care, leading to undertreatment or increased toxicity. With this background, it is crucial to investigate new opportunities that belong to the most recent findings of an anti-cancer agent, such as immune-checkpoint inhibitors, to manage these daily clinical issues and eventually combine them with alternative administration strategies of antiblastic drugs such as metronomic chemotherapy.

## 1. Introduction

Although older cancer patients represent the bulk of clinical oncologic practice, fewer data exist regarding the risk and benefit of cancer treatment in this population, mostly because they are under-represented in most clinical trials that set international guidelines for standard of care [1,2,3]. This problem becomes even more significant in the oldest old population (above 80 years old) and, in general, leads to undertreatment of this category due to inadequate knowledge of tolerability and efficacy of anti-cancer therapy [4].

For these reasons, it is necessary to further explore this specific field to be better aware of the potential treatment planning for older cancer patients, including incorporating them in clinical trials aiming to find their best standard of care and alternative treatment strategies. We reviewed the main available treatment strategies for cancer treatment in the older cancer population, focusing on a potential new treatment approach for this category of patients.

## 2. Epidemiologic and Mechanistic Data

In the United States, approximately 50% of new cancer diagnoses and 70% of cancer-related mortality occurs in patients older than 65 [5]. A similar statistic is observed in Europe, with more than 50% of newly diagnosed tumors in those older than 65 [6]. Indeed, aging is a well-recognized risk factor for developing cancer through several mechanisms [7,8]. Genetic and epigenetic mutations, mitochondrial dysfunction, endocrine- and cytokine-mediated pathways, mutations in aging stem cells, and telomere shortening have been the most studied factors [9,10]. Another mechanism of the relationship between aging and cancer development is immunosenescence [11]. This is characterized by different features, such as a lower number of naïve CD8 T+ and CD4 T+ cells in the peripheral blood due to the involution of structures involved in the immune system [11]. The consequence of these elements reduces the T-cells’ ability to be activated and perform their functions, even in tumor growth control, promoting one of the main hallmarks of cancer cell proliferation, immune escape [12]. 

## 3. Maximum Tolerated Dose of Chemotherapy

Conventional chemotherapy is based on the maximum tolerated dose (MTD) concept, a strategy characterized by the infusion of a chemotherapy drug at the highest dose that presents tolerable side effects. Since the 1960s, this method has been applied to cure hematological malignancies, showing for the first time an efficacious strategy for these diseases. However, the MTD chemotherapy is related to a not negligible series of side effects due to the destruction of a relevant fraction of highly reproducing normal cells, such as those lining the gastrointestinal tract and bone marrow cells [13,14]. 

In general, the cost-effectiveness for toxicity is well balanced in the younger population, especially with the introduction of supportive drugs, such as antinausea or granulocyte-stimulating factors (G-CSF), preventing the most common side effects related to chemotherapy regimens [15]. 

The same outcomes are not available for older cancer patients for many reasons. Aging is associated with a decline in function of end organs. For example, liver function could be compromised by slower drug metabolism and this is related to higher chemotherapy concentration exposure for more extended periods if hepatically cleared [16,17]. The same occurs with renally cleared agents because the glomerular filtration is reduced over time. In the bone marrow, its reserve diminishes with aging, leading the patients to experience prolonged cytopenias [16]. Myelosuppression is not just a quantitative issue of MTD chemotherapy, but it is represented by a “qualitative” issue worthy of being discussed. In particular, high-dose chemotherapy leads to a dysfunction of natural killer (NK) and γδT cells, compromising immune tolerance [18]. Moreover, high-dose chemotherapy can interfere with dendritic cells (DCs), decreasing their antigen-presenting ability, reducing their mobility, and downregulating the expression of cell surface markers. Other issues to consider are that older cancer patients usually are frail, have different comorbidities at diagnosis that could compromise chemotherapy tolerability, and take a great number of drugs that could interfere with anti-cancer treatment [19]. Finally, more and more data on chemotherapy-induced cognitive impairments are being reported in the last decades [20].

All these aspects limit clinicians in treatment decision making, leading to recommendations of best supportive care rather than chemotherapy given the potential risks. Several prediction tools have been developed specifically on this topic, such as: Chemotherapy Risk Assessment Scale for High-Age Patients (CRASH) score, able to stratify the patients in four risk categories of severe toxicity; chemotherapy toxicity calculator from the Cancer and Aging Research Group (CARG score), a predictive model for chemotherapy toxicity in patients ≥65 years old; and Geriatric 8 score (G8), a screening tool to determine which older cancer patients should undergo full geriatric evaluation prior to commencing chemotherapy [21,22,23]. 

## 4. Metronomic Chemotherapy

An alternative approach, low-dose metronomic chemotherapy (MC), has been evaluated in recent years to mitigate the disadvantages of MTD chemotherapy and to try to overcome resistance mechanisms [24]. Its goal is to administer low doses of chemotherapy without interruption and it has been tested in various studies across multiple histologies [25,26,27,28]. 

This new scenario gives many opportunities against cancer cell proliferation and acquired tumor resistance through several mechanisms. The main fascinating feature of MC is its effect on the tumor cell growth pathways and the impact on the tumor microenvironment. In particular, to provide their development, tumor cells need to produce new blood vessels, to support the high energy needs due to their rapid proliferation, a process known as angiogenesis [29]. Vascular endothelial growth factor (VEGF), fibroblast growth factors (FGF), platelet-derived growth factor (PDGF), epidermal growth factor (EGF), and thrombospondin-1 (TSP-1) are all involved in blood vessel genesis. They are produced in a high fraction by tumor cells [30]. In this background, MC has been shown to induce apoptosis of activated endothelial cells and endothelial migration and reduce the activity of essential angiogenesis factors [31,32]. 

Another effect on the tumor cell microenvironment is related to the immune system. As mentioned above, immune escape represents one of the most important and most studied hallmarks for cancer cell proliferation. It consists of the production and stimulation of immunosuppressive molecules in order to silence both the innate and adaptive immune response and avoid tumor infiltration and destruction [33]. 

Several cell types are involved in this challenge, such as Treg cells, able to suppress tumor-specific effectors (CD8+ T lymphocytes, CD4+ T helper cells and NK cells) and myeloid-derived suppressors (MDSC’s), able to suppress T and NK cells through different mechanisms [34,35]. In recent years, some evidence showed that MC could potentiate host immunity by many immunomodulatory mechanisms. For example, the continuous exposure of a low dose of cyclophosphamide showed in vitro to stimulate through the macrophages the secretion of pro-inflammatory factors (IL-6 and IL-12), downregulation of anti-inflammatory cytokines (TGF-β) and IL-10, and reduction of Treg levels [36,37]. Low concentrations of methotrexate, paclitaxel, vincristine, and vinblastine promote maturation of DCs and their antigen-presenting activity [38]. 

The advantages of MC are not just biological, but they play an essential role in clinical aspects. In particular, MC shows a great anti-cancer activity and survival benefit in the main oncologic outcomes, such as overall survival and progression-free survival, in different kinds of histologies, and an excellent profile of safety, especially in older cancer patients [25,26,27,28,39]. The two agents most studied in this setting are capecitabine and vinorelbine. These benefits were kept in the older cancer patients, even when these agents were administered in combination at metronomic dosage [40]. 

Indeed, it is more and more frequent that oncologists choose this strategy to treat those frail older patients unable to tolerate a standard chemotherapy regimen. 

## 5. Immunotherapy

The antitumoral role of the immune system has been known since William Coley discovered that the injection of inactivated bacteria into sites of sarcoma could lead to tumor shrinkage [41].

Several kinds of immunotherapies have been under investigation in recent years. The manipulation of immune checkpoints, in particular cytotoxic T-lymphocyte antigen 4 (CTLA-4) and programmed cell death 1 (PD-1), known as an immune checkpoint inhibitor (ICI), has been of particular interest in this regard [42]. 

These transmembrane proteins are expressed on immune system cells: CTLA-4 on the cell surface of CD4+ and CD8+ T lymphocytes, PD-1 on the surface of T cells, B cells and NK cells. All of these are able to activate inhibitor pathways in these cells and a transformation in immunosuppressive landscape, such as with Treg cells for instance [43,44,45]. Blockade of these inhibitory cell surface proteins shows excellent efficacy in many malignancies, such as renal cell carcinoma, melanoma, non-small cell lung cancer, and breast cancer, with others still being further elucidated [46,47,48,49]. 

These results are also confirmed in the older population and represent a valid treatment strategy, even in the oldest old population. The improvement in survival associated with ICIs is consistent across an age cut-off of ≥65 years old. However, more data are needed to understand these results among the oldest old patients (>75 years) [50,51]. This approach offers a frequently tolerable option given that these drugs have a variable side effect profile but are more often than not very well tolerated even in patients with a compromised performance status.

## 6. Metronomic Chemotherapy and Immunotherapy: A New Horizon?

In older individuals requiring combination chemo-immunotherapy, a new model of anti-neoplastic treatment could be combination of low-dose metronomic chemotherapy and immunotherapy. There are data suggesting that certain cytotoxic agents could enhance the efficacy of immunotherapy and further data outlining the immunostimulatory potential of metronomic therapy. Several recent preclinical studies have explored this field outlining promising results [52,53]. 

In one trial, 28 metastatic melanoma patients with progressive disease were treated with a metronomic dose of cyclophosphamide (50 mg twice a day for 1 week altering with off treatment) and celecoxib (200 mg daily throughout the study) followed by vaccination with DCs, showing improved survival compared to retrospective data of treatment without chemotherapy and celecoxib [54]. Encouraging results were found even with the new class of ICI drugs. Karachi et al. demonstrated a relationship between peripheral and tumor immune microenvironment transformation and dose modulation of temozolomide in murine models [55]. Moreover, some data show that the anti-PD1 activity dampens glycolysis, providing cytotoxic lymphocytes with an additional competitive advantage [56] (Table 1 and Table 2).

In summary, both MC and immunotherapy seem to increase immune cell activation, the former promoting tumor-specific activation and the latter maintaining that activation. MC can affect the tumor microenvironment and facilitate tumor infiltration and cytotoxic effects [53]. 

This evidence outlines an encouraging option via the synergistic effect of low-dose metronomic chemotherapy and immunotherapy, allowing efficacious treatment while minimally affecting quality of life in frailer patients and opening up a potential option in those that may have just had supportive care offered. 

## 7. Final Remarks

Anti-neoplastic treatment in older patients is still problematic since this population is under-represented in clinical trials. The scientific community should stress the importance of conducting clinical trials to assess treatment efficacy and safety in geriatric populations. The management of these patients should be multidisciplinary with the involvement of disease specialists and geriatricians in order to have these patients appropriately evaluated with validated tools that can better predict how they will tolerate treatments and how effective these treatments might be.

Data in the older population have shown that toxicities often do not allow the same dose intensity as in younger patients and the vast majority of geriatric patients will receive less effective and sometimes still toxic treatments.

This leads to conventional chemotherapy regimens, both monotherapy and combination regimens, being modified in their schedules and in reduced dose intensities reducing efficacy.

To overcome this issue, recent advances have been made showing how the combination of “new” and “old” therapies, such as immunotherapy with ICIs and MC, might be an option to avoid cancer progression and resistance. Emerging data have been encouraging but also very premature since they derive from little clinical experiences or in vitro models. There is an urgent need for such approaches to be validated in wider, placebo-controlled, randomized trials conducted on the older population in concert with appropriate clinical evaluation through validated geriatric tools. The strong preclinical rationale and the favorable toxicity profile seem to be the winning step of this anti-cancer therapy combination. It is crucial to keep on studying these approaches in clinical practice. 

## Figures and Tables

**Table 1:** Synergistic effect of immunotherapy and metronomic therapy. Preclinical data.

Author, Year Reference	Study Design	Treatment Regimen	Outcomes
Xu C et al., 2017	EMT-6 and MC38 murine tumor models	NHS-muIL12 (2 or 10 μg) and avelumab (200 μg) combination therapy	Complete tumor regression, generation of long-term tumor-specific protective immunity
Ma J, 2009	PC-3 tumors and 9 L gliosarcoma xenograft models in mice	Axitinib (25 mg/kg body weight) and cyclophosphamide (140 mg/kg body weight) combination therapy	Increased antitumor activity
Ko H.-J, 2007	Tumor model in mice; mouse Her-2/neu as self-antigen investigated whether genetic vaccination with DNA plasmid and/or adenoviral vector expressing the extracellular and transmembrane domain of syngeneic mouse Her-2/neu or xenogenic human Her-2/neu could induce mouse Her-2/neu–specific CTL responses.	60 mg/kg (1.2 mg per mouse) of gemcitabine, followed by AdhHM ^1^ treatment and agonistic αGITR Ab ^2^ (DTA-1 ^3^)	Higher levels of therapeutic antitumor immunity
A Karachi, 2019	GL261 and KR158 murine glioma models	MD ^4^ temozolomide (25 mg/kg × 10 days) or standard dose temozolomide (50 mg/kg × 5 days) and PD-1 antibody combination therapy	Decrease in exhaustion markers in tumor infiltrating lymphocytes with MD temozolomide/PD-1 antibody group Benefit of PD-1 inhibition’s reduction with standard dosing strategies of temozolomide

^1^ Adenoviral vectors expressing xenogenic human Her-2/neu. ^2^ Agonistic anti-glucocorticoid-induced TNFR family-related receptor (GITR) antibody. ^3^ anti-GITR. ^4^ Metronomic dose.

**Table 2:** Synergistic effect of immunotherapy and metronomic therapy. Clinical data.

Author, Year	Study Design	Primary Endpoint	Study Population, *n*, Disease, Mean Age (Range)	Treatment Regimen	Outcome
M Podrazil, 2015	Open-label, single-arm Phase I/II clinical trial	Safety and immune responses	25 patients, metastatic CRPC ^a^, median age 73 years (age range 48–82)	Docetaxel combined with treatment with autologous mature DCs ^b^ pulsed with DCVAC/PCa ^c^ Twelve doses of 1 × 10^7^ dendritic cells injected. Administration of docetaxel (75 mg/m^2^) and prednisone (5 mg twice daily) after the initial two doses of DCVAC/PCa Metronomic cyclophosphamide (Cyclophosphamide Orion^®^ 50 mg daily for 1 week), before the 1st DCVAC/PCa dose	PSA reduction by at least 50% on two visits at least 6 weeks apart in 39.1% A ≥50% decrease in PSA in 34.8% of patients at 6 months after the initiation of chemo/immunotherapy Median survival of 19 months for the DCVAC/PCa-treated group compared to 11.8 months in the Halabi and 13 months in the MSKCC control predictions Improvement in the median overall survival with combined docetaxel and DCVAC/PCa
N Abd El Bary, 2010		Tolerability and PFS	41 patients, istologically aggressive non-Hodgkin lymphoma, median age 56 years	Oral cyclophosphamide (50 mg every day), oral methotrexate (2.5 mg 4 times/week) and high-dose oral celecoxib (400 mg twice daily) until disease progression or unacceptable toxicity	No major toxicities Partial response in 31.7% Stable disease in 48.8% Progression-free survival was 12 months Median response duration was 10 months
E Ellebaek, 2012	Phase II vaccination trial	Tolerability and safety, immunological and clinical response and to determine PFS as well as OS	28 patients, progressive metastatic melanoma, median age 58 years (age range 22–82)	Metronomic dose of cyclophosphamide (50 mg twice a day for 1 week altering with off treatment) and celecoxib (200 mg daily throughout the study) followed by vaccination with DC	Improved survival compared to treatment without chemotherapy and celecoxib Prolonged survival in SD ^d^ patients compared with PD ^e^ patients (10.5 vs. 6.0 months, *p* = 0.048)

^a^ Castration-resistant prostate cancer. ^b^ Dendritic cells. ^c^ Killed LNCaP prostate cancer cells. ^d^ Stable disease. ^e^ Progressive disease.

## Data Availability

Not applicable.
